# Weibo Users’ Emotion and Sentiment Orientation in Traditional Chinese Medicine (TCM) During the COVID-19 Pandemic

**DOI:** 10.1017/dmp.2021.259

**Published:** 2021-08-09

**Authors:** Hao Gao, Difan Guo, Jing Wu, Lina Li

**Affiliations:** 1 School of Journalism and Communication, Nanjing Normal University, Nanjing, China; 2 Faculty of Social Sciences, University of Ljubljana, Ljubljana, Slovenia; 3 Film-Television and Communication College, Shanghai Normal University, Shanghai, China

**Keywords:** Traditional Chinese Medicine (TCM), COVID-19, sentiment dictionary, emotion, social media

## Abstract

**Objective:**

This study aimed to explore Chinese people’s attitudes to the official application of TCM in coronavirus disease 2019 (COVID-19) treatment.

**Methods:**

We collected data referring to TCM on Weibo from 0:00 on January 24, 2020, to 23:59:59 on March 31, 2020 (Beijing time). In addition, this study used DLUT-Emotion ontology to analyze the sentiment orientation and emotions of selected data and then conducted a text analysis.

**Results:**

According to DLUT-Emotion ontology, we examined 3 sentiment orientations of 215,565 valid Weibo posts. Among them, 25,025 posts were judged as positive emotions, accounting for approximately 12%; 22,362 were regarded as negative emotions, accounting for approximately 10%; and 168,178 were judged as neutral emotions, accounting for approximately 78%. Results indicate that the words judged as “Good” have the highest frequency, and words marked as “Happy” have increased over time. The word frequency of “Fear” and “Sadness” showed a significant downward trend.

**Conclusion:**

Weibo users have a relatively positive attitude to the TCM in the COVID-19 treatment in general. Results of text analysis show that data with negative emotions is essentially an expression of public opinions to supporting TCM or not. Texts of “Fear” and “Sadness” do not reflect users’ negative attitudes to TCM.

Coupled with its long history and long-term impact in China, Traditional Chinese Medicine (TCM) is an essential component of the Chinese medical industry. Some researchers found that too much “fake TCM” information misled the public after severe acute respiratory syndrome (SARS) in 2003, which caused public prejudice against TCM.^1^ In the early stage of the outbreak of coronavirus disease 2019 (COVID-19) in China, the National Health Commission of the PRC officially wrote the TCM application into COVID-19 treatment protocols. Since then, hot discussion about TCM was triggered on social media, including Sina Weibo. As an open platform for expressing opinions, Sina Weibo is a source of understanding the publics’ ideas and attitudes. To explore the public attitudes to the TCM application into the COVID-19 treatment, we researched the relevant discussion and topics on Sina Weibo. This study used emotion and sentiment analysis to analyze the public opinions expressed on Sina Weibo, answering 2 research questions:

RQ1: From the TCM-related information posted on Weibo during the COVID-19, what sentiment orientation and specific emotions do the public show regarding the TCM application into the COVID-19 treatment?

RQ2: What kinds of public opinions and attitudes toward TCM are reflected in sentiment orientation and specific emotions?

## Methods

### Data Collection

To examine the public attitudes to TCM shown on Sina Weibo, we collected data referring to TCM on Weibo from 0:00 on January 24, 2020, to 23:59:59 on March 31, 2020 (Beijing time). We determined the start date of the data as when Hubei Province launched its first-level response to major public health emergencies,^2^ and selected the end date of the data regarding when the Central Steering Group claimed that the spread of the national epidemic had been stopped.^3^ A total of 215,565 valid posts were obtained through data cleaning, with 5,642,598 reposts.

### Emotion and Sentiment Analysis

Considering the low granularity and short words of Weibo posts, this study used DLUT-Emotion ontology to analyze the sentiment orientation and emotions of selected data. DLUT-Emotion ontology refers to authoritative Chinese dictionaries such as A Thesaurus of Modern Chinese, A Dictionary of Chinese Praise and Blame Words, and Chinese Dictionary of Adjectives, containing nearly 30,000 emotion words. With high coverage and accuracy, the tool is widely used in Chinese sentiment analysis.^4^

DLUT-Emotion ontology defines the sentiment polarity of words as positive, neutral, and negative words. Furthermore, the tool labels sentiment intensity of words with 5 levels (1, 3, 5, 7, 9), a higher value means stronger sentiment intensity. In addition, DLUT-Emotion ontology marks each word with sentiment attributes according to the 7-layer-division method, which was developed by adding “good” to Ekman’s 6 basic emotions. Table 1 gives a detailed description of how DLUT-Emotion ontology defining words with sentiment orientation and attributes.

**Table 1.** Examples of words defined with sentiment orientation and attribute in DLUT-Emotion ontology

Class	Item	Sample words
Sentiment polarity	Positive	Confidence (5), outstanding (5), cure (9), efficacy (3), advantage (5), elite (9), thanks (1), fair (9), fit (1), reliable (7), calm (3), reasonable (1), innovation (3)
	Neutral	suggestion (5), tips (7), reason (5), safe (7), strong (1), desperately (9), outrage (9), rare (3), interest (3), clear (3), relax (1), transient(1)
	Negative	inefficiency (5), tense (5), pressure (5), desperate (9), fraud (5), unfair (3), fake (3), incompetent (5), profit-making (3), buy off (7), sinister (9), unbearable (9)
Sentiment attribution	Good	advanced, excellent, profound, trust, respectful, great, strictly, omnipotent, outstanding, zeal, agree, admire
	Happy	happy, inspire, joy, smilingly, dependable, delightful, expectation, achievement, relaxed, high, carefree, excited, recover, fun, resolve
	Surprise	strange, miracle, shocked, surprising, extremely, suddenly, rare, faint, everywhere, happen, rise
	Disgust	stupid, question, lie, exaggerate, shameful, hate, vanity, rigid, harm, hostility, despise, curse, hypocrisy, dirty, stoop, narrow, betray, crafty
	Fear	tense, salvage, inefficiency, disease, flustered, afraid, helpless, twisty, haunted, blankly, cramped, change, towering, critical, disease
	Anger	angry, furious, charlatan, peeved, fury, blame, rail, afflicted, protest, dare, quarrel, reproach, revenge, stare, punish, roar
	Sadness	mourn, painful, helplessness, cry, distressed, guilt, depressed, miss, loneliness, lose, poverty, disaster, murdered, pity, melancholy, failure, suffering, victim

**Table 2.** Results of sentiment orientation and attribute

	Sentiment orientation	Volume	%	Sentiment attribute	Volume	%
Stage 1 (Jan. 24 - Feb.1)	Positive	8087	9.58%	Good	111657	51.51%
				Happy	5234	2.41%
	Neutral	63934	75.77%	Surprise	732	0.34%
				Disgust	34359	15.85%
	Negative	12365	14.65%	Fear	54640	25.21%
				Anger	454	0.21%
				Sadness	9672	4.47%
Stage 2 (Feb.2 - Feb. 14)	Positive	17474	14.59%	Good	138876	62.93%
				Happy	20736	9.40%
	Neutral	91395	76.29%	Surprise	645	0.29%
				Disgust	33978	15.40%
	Negative	10924	9.12%	Fear	17961	8.14%
				Anger	479	0.22%
				Sadness	7994	3.62%
Stage 3 (Feb.15 - Mar. 31)	Positive	13352	15.50%	Good	123679	58.10%
				Happy	51998	24.43%
	Neutral	67527	78.42%	Surprise	695	0.33%
				Disgust	16013	7.52%
	Negative	5237	6.08%	Fear	14039	6.59%
				Anger	440	0.21%
				Sadness	6006	2.82%

In terms of data processing, the computer matches the words contained in the selected data with DLUT-Emotion ontology and determines the sentiment orientation of each data sample based on the sentiment polarity and sentiment intensity of words. The sentiment attributes of each data sample were defined according to the 7-layer division of DLUT-Emotion ontology.^5^ The results indicate the Weibo users’ sentiment orientations and attributes to the TCM application in COVID-19 treatment.

### Text Analysis

In addition to the sentiment orientation and attributes analysis, this study conducted a text analysis to explore the specific connotation of expressed emotions and sentiments, which can reflect the specific attitudes of Weibo users to the TCM application in COVID-19 treatment.

## Results

### Results of Emotion and Sentiment Analysis

According to DLUT-Emotion ontology, we examined 3 sentiment orientations of 215,565 valid Weibo posts. Among them, 25,025 posts were judged as positive emotions, accounting for approximately 12%; 22,362 were regarded as negative emotions, accounting for approximately 10%; and 168,178 were considered neutral emotions, accounting for approximately 78%.

We found that the public sentiment orientations changed over time: Weibo texts with negative emotions weighed more than positive emotions from January 24 to February 1; the proportion of Weibo texts with negative emotions began to decline since February 2, while positive texts gradually increased and finally overshot until March 30; the wave of both texts with negative and positive emotions tended to be stable since February 15.

According to the findings, this study divided selected Weibo texts into 3 periods: January 24 to February 1, February 2 to February 14, and February 15 to March 30. The calculation results showed that words judged as “Good” have the highest word frequency; words regarded as “Fear” and “Happy” varied considerably among the 3 periods. The word frequency of “Fear” showed a significant downward trend while “Happy” appeared upward after the first period.

## Discussion

### Relatively Positive Attitudes to TCM in General

From the results of sentiment orientation analysis, Weibo users have a relatively positive attitude to the TCM in the COVID-19 treatment in general. Twelve percent of collected data indicated positive emotions, 10% showed negative emotions, and 78% were neutral. The text analysis further analyzed the public attitudes reflected by different sentiment orientations.

Among the data judged as positive emotions, keywords of these Weibo posts are “diagnosis,” “severe,” “clinical,” “participation,” “discharge,” “effective,” and “cure,”, which focused on the topic of the outstanding achievements of TCM in treating COVID-19. Data related to this topic mainly consisted of 2 aspects. On the one hand, Weibo users repost media releases affirming the effectiveness of TCM in treating COVID-19. On the other hand, users post original content expressing their affirmation of the efficacy of TCM in COVID-19 treatment and the pride of TCM, such as “I am proud of our TCM.” Previous research claimed that the Chinese public preferred expressing their opinions and views by means of social media like Sina Weibo.^6^ In terms of the 12% positive content publishers, most of them are TCM supporters. They held a positive attitude toward TCM and promoted it on Weibo as TCM was effective in anti-pandemic.

Keywords of the data marked as neutral emotions are “health preservation,” “TCM doctors,” “take care,” “acupoints,” “acupuncture,” “spleen and stomach,” “health,” “tips,” and “effective.” These Weibo data are around the topic of daily health preservation with TCM, and most of the data are the repost of TCM-related information. The contents involve TCM health care, TCM application in daily life, and TCM folk prescription for treating diseases. Hospitals and other medical institutions released the origins of these Weibo posts. A study of motivations for sharing information on social media showed that Twitter users would like to share helpful information.^7^ Weibo users forwarding TCM-related information on social media indicated that they trust and support TCM, and they also advocate the use of TCM for health management.

Text analysis of data with negative emotions found 2 different attitudes among the public, and they hold opposite opinions on TCM. On the one hand, some users questioned TCM, especially for the scientific evidence of the TCM application in COVID-19 treatment. For example, they post as “whether the efficacy of TCM pneumonia prevention agents recommended by Hubei Daily and Wuhan Tongji Hospital has been verified.” On the other hand, although negative emotional words were used in the texts, these words were not aimed at TCM. This kind of text shows the public support to TCM essentially. The texts were against ideas and opinions of belittling and misunderstanding TCM, such as “I trust Western medicine more in disease diagnosis and surgery, but I do not reject Chinese medicine, especially in the aspect of body healing. I do not understand why people view Chinese medicine and Western medicine as opposites.” In addition, some of the texts reflected the concerns about the education and inheritance of TCM. For example, “Why is it so difficult to develop TCM? Because there is no successor.” Users expressed their opinions on Weibo with their original creation and formed a battle of “supporting TCM” and “questioning TCM” online. It is also in line with the nature of Sina Weibo as social media, which provides a communication space for different discourses.^8^

### Changes in Specific Emotional Connotations

From the perspective of the changes in specific emotional connotation, changes of positive emotions reflect people’s support and affirmation to TCM, and changes of negative emotions indicate the change of people’s perception of the epidemic.

According to sentiment analysis, the words judged as “Good” have the highest frequency, and words marked as “Happy” have increased over time. Texts related to “Good” and “Happy” were about the praise of TCM achievements in COVID-19 treatment, affirmation of TCM advantages in prevention, the pride of having TCM as a treasure of Chinese culture, and appreciation for TCM medical workers. Hence, the increase of “Good” and “Happy” words after the control of the epidemic reflected the recognition of TCM by Weibo users.

In addition, the frequency of words defined as “Sadness” and “Fear” decreased over time. Through the text analysis of sentences marked as “Sadness,” we found that when TCM and sadness words appear simultaneously, the sentence would be defined as “Sadness”. For example, “I marveled at the selflessness of TCM workers that they sacrificed their lives for us,” which reflected the grief and mourning of netizens over the death of TCM workers due to the epidemic, rather than their attitudes to TCM. Furthermore, when users use words like “tense” and “terrible” with “TCM” and “COVID-19” in the exact text, the text would be determined as “Fear.” For instance, “We are struggling with the virus tensely, should TCM community start to develop a sovereign remedy for against COVID-19,” which pointed out a “Fear” of the virus rather than TCM. It is reasonable that people’s fear of the disease has been reduced with the control of the pandemic, and the frequency of “Fear” words decreased. A survey of the mental health under the pandemic in the United Kingdom also proved that strong public health measures could reduce the public worries about being infected and significantly improve public mental health.^9^

## Conclusions

With the emotion and sentiment analysis based on big data, this study analyzed the sentiment orientation and attributes contained in Weibo texts about the TCM application in treating COVID-19. From the text analysis of positive and neutral texts, as well as the connotation of positive emotions like “Good” and “Happy,” Weibo users generally recognize and accept the application of TCM in treating COVID-19. Results of text analysis show that data with negative emotions is essentially an expression of public opinions to supporting TCM or not. Texts of “Fear” and “Sadness” do not reflect users’ negative attitudes to TCM.

Emotion and sentiment analysis is a method to evaluate the citizens’ opinions and attitudes in the era of big data. However, this method has certain limitations that we need to combine with qualitative methods like text analysis to avoid evaluation deviations. In addition, the results of this study only reflect the public attitudes during specific periods, and further monitoring is needed. However, analyzing social media texts is helpful to get a snapshot of public views. It is also beneficial to understand the public attitudes to TCM, carry out targeted dissemination of TCM-related issues, and promote the general cognition of TCM health issues.
